# Effect of CGRP inhibitors on interictal cerebral hemodynamics in individuals with migraine

**DOI:** 10.3389/fneur.2024.1399792

**Published:** 2024-04-29

**Authors:** Sarah C. Carter, Brett Cucchiara, Navpreet Reehal, Katherine Hamilton, Eric A. Kaiser, Christopher G. Favilla

**Affiliations:** Department of Neurology, University of Pennsylvania, Philadelphia, PA, United States

**Keywords:** calcitonin gene-related peptide, chronic migraine, CGRP inhibitor, cerebral autoregulation, cerebral hemodynamics, cerebrovascular reactivity

## Abstract

**Introduction:**

Calcitonin gene-related peptide (CGRP) plays an important role in cerebral vasodilation, so here we aim to quantify the impact of CGRP monoclonal antibody (mAb) therapy on cerebral hemodynamics.

**Methods:**

In 23 patients with chronic and episodic migraine, cerebral hemodynamic monitoring was performed (1) prior to and (2) 3-months into CGRP-mAb therapy. Transcranial Doppler monitored cerebral blood flow velocity (CBFv) in the middle cerebral artery (MCA) and posterior cerebral artery (PCA), from which cerebrovascular reactivity (CVR) and cerebral autoregulation (CA; *Mx-index*) were calculated.

**Results:**

CA was similar off and on treatment, in the MCA (*p* = 0.42) and PCA (*p* = 0.72). CVR was also unaffected by treatment, in the MCA (*p* = 0.38) and PCA (*p* = 0.92). CBFv and blood pressure were also unaffected. The subgroup of clinical responders (>50% reduction in migraine frequency) exhibited a small reduction in MCA-CBFv (6.0 cm/s; IQR: 1.1–12.4; *p* = 0.007) and PCA-CBFv (8.9 cm/s; IQR: 6.9–10.3; *p* = 0.04).

**Discussion:**

Dynamic measures of cerebrovascular physiology were preserved after 3 months of CGRP-mAb therapy, but a small reduction in CBFv was observed in patients who responded to treatment. Subgroup findings should be interpreted cautiously, but further investigation may clarify if CBFv is dependent on the degree of CGRP inhibition or may serve as a biomarker of drug sensitivity.

## 1 Introduction

Migraine is one of the most common causes of disability, affecting more than a billion people globally (1). Calcitonin gene-related peptide (CGRP) is an important therapeutic target in migraine, because of its role in modulating peripheral and central projections of the trigeminovascular system, which facilitates nociception and neurogenic inflammation (2). Inhibition of this pathway, either of CGRP or its receptor, is effective both for migraine prevention and as abortive treatment for migraine attacks. Neurons projecting to the cerebral and peripheral vasculature also secrete CGRP, where it has a potent vasodilatory effect (3, 4).

In healthy brain, CGRP plays a role in cerebral autoregulation (5), with intravenous administration of CGRP resulting in cerebral vasodilation (6, 7) and CGRP antagonism blunting autoregulatory function (8). In the context of cerebral ischemia, CGRP may counteract low perfusion pressure and high vascular tone (9), while CGRP antagonism inhibits collateral flow involved in ischemic injury defense (9). Based on particle size, it has been suggested that anti-CGRP antibodies would not be expected to cross the blood–brain barrier (10, 11), and thus direct CNS or cerebrovascular consequences would be unlikely. However, anti-CGRP antibodies have been shown to inhibit cerebrovascular dilation, raising the possibility of either an indirect effect or action within the vessel wall (12).

In the peripheral vasculature, CGRP also plays an important role in maintaining systemic vascular tone (13). It is implicated in the prevention of onset of hypertension through compensatory vasodilation of small arteries, which modulates peripheral vascular resistance (PVR) (14). Additionally, dose-dependent increases in circulating CGRP levels were observed during infusion of the vasopressor angiotensin II (15), indicating CGRP is released systemically in response to acutely increasing blood pressure, further suggesting an important compensatory role.

Clinical trials of CGRP inhibitors in migraine have generally demonstrated a favorable safety profile to date, with no reported neurovascular complications (16–19). However, patients with cardiovascular or cerebrovascular disease were largely excluded from these trials. Further, trial follow-up was designed to quantify migraine outcomes and thus relatively short-term; this may not be sufficient to exclude longer-term or infrequent vascular adverse events. Given the potential impact of CGRP inhibitors on cerebral hemodynamics, and the implications this might have on vascular risk, we aimed to investigate cerebral hemodynamics before and during CGRP monoclonal antibody treatment.

## 2 Materials and methods

### 2.1 Participants

Patients were recruited from the outpatient Neurosciences Center at the Hospital of the University of Pennsylvania. Eligible participants were at least 18 years or older, diagnosed with migraine with or without aura, and were newly prescribed long-acting CGRP monoclonal antibody (mAb) therapy for migraine prevention. During the course of the study, this included galcanezumab, fremanezumab, and erenumab. Patients were excluded if they had previously used any CGRP-targeting medication. Additional exclusion criteria included a history of stroke, cerebral vascular abnormality, cerebral mass lesion, and skull defect or prior surgery which could interfere with transcranial Doppler (TCD) monitoring over the temporal region. At the time of enrollment, a case report form captured participant demographics, medical history, migraine frequency, and concurrent medication use. Biological sex is reported in participant demographics as it is recorded within the electronic medical record. In this cohort, biological sex and gender identity were aligned in all subjects. Migraine frequency and concurrent medications were again assessed during the follow-up evaluation, and patients were categorized as responders if migraine frequency was reduced >50%. Structural neuroimaging was not performed within the context of this study, but previously obtained magnetic resonance imaging results were abstracted from the health record if available. Pathologic findings were reported, but a limited number of white matter hyperintensities were considered non-pathologic if the treating neurologist considered them as such.

All study procedures were approved by the University of Pennsylvania Institutional Review Board (IRB #848535), conformed to the principles outlined by the Declaration of Helsinki and STROBE guidelines for observational research. All patients provided written informed consent prior to initiation of study procedures. The data that support the findings of this study will be made available by the corresponding author upon reasonable request.

### 2.2 Hemodynamic monitoring

Monitoring sessions were conducted in the outpatient Neurosciences Center at the Hospital of the University of Pennsylvania. The hemodynamic monitoring protocol, as described below, was performed twice. The first session was performed at the time of study enrollment and prior to the first administration of CGRP monoclonal antibody therapy. The second session was performed after 3–4 months of dosing the CGRP inhibitor as indicated. All participants were studied in the supine position with the head-of-bed elevated to 45°. Clinic rooms were quiet and temperature controlled (23°C). Cerebral blood flow velocity (CBFv) was measured using a Multigon Industries^®^ (Elmsford, NY) Robotic TCD. A 2 MHz probe was secured over the left temporal window to insonate the middle cerebral artery (MCA) and posterior cerebral artery (PCA). Each vessel was confirmed by their characteristic depth ranges, Doppler signal, direction, and velocities. The CBFv waveform from each vessel was recorded for 5 min. The mean flow velocity and pulsatility index (PI) were recorded continuously for each vessel.

A finger plethysmograph system (Finapres^®^ NOVA, Finapres Medical Systems) was secured to the wrist and third digit to provide a continuous non-invasive measurement of the arterial blood pressure (ABP). An inflatable brachial cuff was placed on the same arm to calibrate the Finapres^®^ NOVA prior to data collection. Re-calibration was performed before TCD data collection for each cerebral vessel. Peripheral vascular resistance (PVR), as calculated by the Finapres^®^ NOVA, was also recorded throughout the monitoring session. Finapres and TCD data (waveform and beat-to-beat mean values) were synchronized and recorded at 125 Hz.

### 2.3 Cerebral autoregulation

Cerebral autoregulation (CA) was quantified by the mean velocity index (Mx index), which represents the correlation of CBFv and ABP (20, 21). CBFv and ABP are averaged over non-overlapping 3-s blocks, and a correlation coefficient is calculated for each minute (i.e., each 1 min epoch contains 20-blocks). Mx index is defined as the average of the correlation coefficients during the monitoring period. Prior to performing this calculation, raw waveform data were visually inspected for artifacts, which were manually removed if present. Three-second blocks were omitted if >50% of a block was missing due to artifact. One-minute epochs were omitted if >50% of blocks within a given epoch were missing due to artifact.

### 2.4 Cerebrovascular reactivity

Cerebrovascular reactivity (CVR) was quantified by comparing CBFv before and after a hypercapnic stimulus. With the TCD in place, an anesthesia facemask (ClearLite, Intersurgical Inc., East Syracuse, NY) was placed over the participant’s nose and mouth. The facemask was connected to a breathing circuit (Teleflex^®^, Wayne, PA) which was capable of delivering either room air or 5% CO_2_ (21% O_2_, Balance N_2_; Airgas^®^, Radnor, PA). A Philips Lo Flow sidestream capnometer (Philips Medical Systems, Andover, MA) was integrated in the respiratory circuit to monitor end-tidal CO_2_, which was synchronized with the CBFv and ABP data. One minute of baseline data was collected while the patient was breathing room air, after which 5% CO_2_ was administered for 2 min, at 8–10 liters per minute. Then, CO_2_ was stopped and the patient reverted to breathing room air. CBFv, ABP, and end-tidal CO_2_ were continuously collected throughout the duration of the challenge. CVR was calculated as:

$$CVR=\frac{CBFv_{\mathrm{final}}-CBFv_{\mathrm{initial}}}{CBFv_{\mathrm{initial}}}x\frac{100}{\Delta CO_{2}}$$

*CBFv initial* was the average of the 1-min baseline period and *CBFv final* was the average of a 10-s epoch that represented the participant’s maximum CBFv during hypercapnia.

### 2.5 Statistics

Primary outcomes included both change in CA (Mx index) and change in CVR between baseline and follow up. Summary statistics were presented as proportions for categorical variables, means (standard deviation) for normally distributed continuous variables, or medians (interquartile range) for non-normally distributed continuous variables. Baseline and follow-up hemodynamic parameters were compared using paired t-tests or Wilcoxon signed rank sum tests, as appropriate. Univariate models evaluated the relationship between the co-primary outcomes and demographics, medical comorbidities, and blood pressure, after which any significant variables were included in a multivariate model along with age and sex. The change in hemodynamic parameters was compared between responders (>50% reduction in migraine frequency) and non-responders by Wilcoxon-Mann–Whitney tests. Baseline characteristics were also compared between responders and non-responders by Wilcoxon-Mann–Whitney test or Fisher’s exact test for continuous and categorical variables, respectively. A 20-patient cohort provides 80% power (setting alpha to 0.05) to detect a change in CVR of 0.5, assuming a baseline CVR of 4 (and standard deviation of 1). This sample size also provides 80% power to detect a change in Mx index of 0.05, assuming a baseline Mx index of 0.30 (and a standard deviation of 0.10). All statistical analyses were performed in STATA/SE version 15.1 (StataCorp LLC, College Station, TX).

## 3 Results

We enrolled and completed the baseline evaluation in 23 patients. Subsequently, 2 patients did not start CGRP monoclonal antibody therapy, 1 discontinued therapy after a single dose due to side-effects, and 1 was lost to follow-up. Nineteen patients completed the follow up evaluation. One patient was excluded from analysis due to inadequate TCD insonation. Thus, 18 patients were included in the analysis. All continued to receive monthly monoclonal antibody therapy at the time of final study visit: 11 taking fremanezumab, 7 galcanezumab, and 1 erenumab. The follow-up monitoring session was conducted after a median of 3 (IQR: 3–4) monthly doses and was a median of 9 days (IQR: 5–21) following the most recent dose. Patient demographics and baseline characteristics are summarized in Table 1. The majority of participants carried a diagnosis of chronic migraine, though the median number of reported headache days in the month prior to enrollment was 15 (IQR: 10–30). This improved to 11 (IQR: 6–15) at the time of the 90-day evaluation (*p* = 0.003). With respect to prior neurologic history, 3 patients had a history of mild traumatic brain injury (TBI) or concussion, one had a history of remote severe TBI, and one had a history of idiopathic intracranial hypertension which resolved several years prior to enrollment. Clinical neuroimaging data were available for 13 of 18 patients, the majority of which were unremarkable (Table 1). Concomitant medication use was summarized in Supplementary Table S1. Overall, there was a paucity of potentially vasoactive medications within the cohort. Importantly, the daily medications listed in Supplementary Table S1 were unchanged between the two study visits. The only noted medication difference (other than the CGRP monoclonal antibody) was that 2 patients used a triptan during the 48 h preceding the first study visit, only one of whom also used a triptan preceding the second study visit.

**Table 1:** Demographics and baseline medical history.

	Cohort *n* = 18
Age, years	42.3 (14.7)
Sex, % female	72%
Race, %	
White	83%
Black or African American	11%
Asian	6%
Migraine characteristics	
Baseline migraine frequency, n/month	15 (10–30)
Diagnostic classification, % with chronic migraine	94%
Migaine with aura	33%
Lifetime burden, years	15 (9)
Medical history	
Hypertension	11%
Type-2 diabetes	6%
Hyperlipidemia	17%
Coronary artery disease	0%
Heart failure	0%
Cigarette smoking, current	6%
Mild TBI or concussion	17%
Severe TBI	6%
Idiopathic intracranial hypertension, resolved	6%
Neuroimaging findings	
No pathologic findings	85%
Pineal cyst	8%
Diffuse white matter hyperintensities of unclear etiology	8%

Normally distributed continuous variables are reported as mean (standard deviation). Non-normal or ordinal variables are reported as median (interquartile range). Categorical variables are reported as proportions. TBI indicated traumatic brain injury. Neuroimaging data were available for 13 of 18 patients, so proportions were reported relative to the 13 for whom data were available.

**Figure 1:**
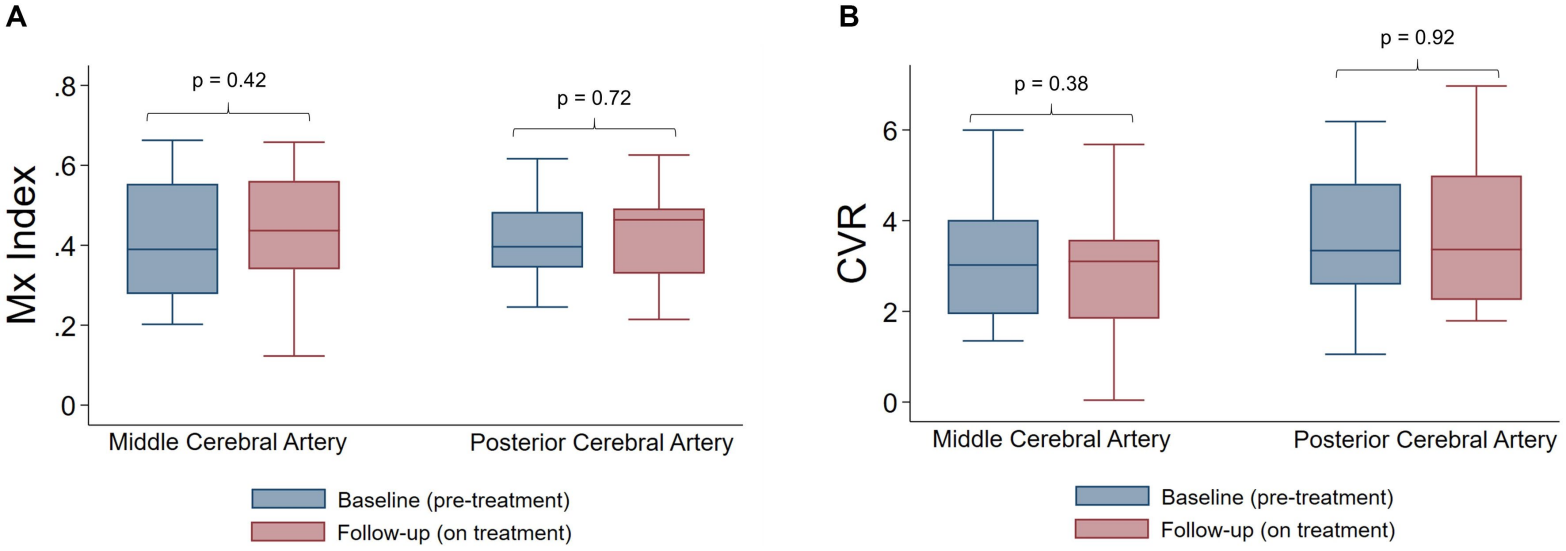
Autoregulation and cerebrovascular reactivity before and after CGRP targeted therapy. **(A)** Mx index is not significantly different pre-treatment and on treatment, in both the middle cerebral artery and posterior cerebral artery. **(B)** Cerebrovascular reactivity is not significantly different pre-treatment and on treatment, in both the middle cerebral artery and posterior cerebral artery. *p*-values were calculate by Wilcoxon signed rank sum tests. CVR indicates cerebrovascular reactivity.

With respect to primary outcome measures, on-treatment follow up evaluation showed no significant change in CA (Figure 1A) or CVR (Figure 1B), as compared to baseline. Overall, no significant changes in systemic or cerebral hemodynamics were observed with CGRP inhibitor therapy (Table 2), with the exception of the PCA PI, which was higher during the follow-up visit (0.93 vs. 1.00, *p* = 0.003) and may reflect an increase in distal vascular resistance. A univariate regression analysis identified no association between the co-primary outcome measures and demographics, medical comorbidities, or blood pressure (Supplementary Table S2). The stability of CA and CVR persisted after adjusting for age and sex in the pre-specified multivariate model.

**Table 2:** Hemodynamics before and after CGRP mAb.

	Baseline *n* = 18	Follow-up *n* = 18	*p*-value
Mean arterial blood pressure, mmHg	97.8 (14.0)	98.6 (13.2)	0.84
Peripheral vascular resistance, mmHg/L/min	0.65 (0.46–0.91)	0.61 (0.46–0.80)	0.64
CBFv, cm/s			
Middle cerebral artery	51.6 (44.2–55.6)	57.2 (46.8–67.8)	0.27
Posterior cerebral artery	27.5 (23.8–37.6)	29.0 (23.9–32.5)	0.78
Pulsatility index			
Middle cerebral artery	0.85 (0.73–0.92)	0.85 (0.79–0.93)	0.93
Posterior cerebral artery	0.86 (0.72–0.99)	1.00 (0.93–1.15)	0.003

Mean arterial pressure is normally distributed so is reported as mean (standard deviation). All other variables are non-normal so are reported as median (interquartile range). *p*-values were calculated by paired *t*-test or Wilcoxon signed rank sum test for normally and non-normally distributed variables, respectively. CBFv indicates cerebral blood flow velocity.

Patients (*n* = 6) who experienced >50% reduction in migraine frequency (i.e., clinical responders) were more likely to experience a reduction in MCA and PCA velocity and an increase in PCA PI with CGRP antagonism (Table 3). Responders and non-responders had a similar change in CA and CVR, and no differences were noted in demographics or baseline characteristics (Supplementary Table S3).

**Table 3:** Comparing hemodynamics based on clinical response (>50% reduction in frequency).

	Responder *n* = 6	Non-responder *n* = 12	*p*-value
Change in MCA velocity, cm/s	−6.0 (−12.4 to −1.1)	6.6 (1.7 to 15.4)	0.007
Change in PCA velocity, cm/s	−8.9 (−10.3 to −6.9)	1.6 (−5.1 to 15.1)	0.04
Change in MCA PI	0.02 (−0.07 to 0.04)	0.03 (−0.06 to 0.05)	0.92
Change in PCA PI	0.23 (0.18 to 0.29)	0.04 (0.00 to 0.12)	0.02
Autoregulation			
Change in MCA Mx index	0.00 (−0.18 to 0.08)	0.08 (−0.07 to 0.22)	0.26
Change in PCA Mx index	0.03 (−0.07 to 0.10)	0.00 (−0.12 to 0.09)	0.75
Cerebrovascular reactivity			
Change in MCA CVR	0.5 (−0.7 to 1.8)	−0.3 (−1.6 to 1.8)	0.60
Change in PCA CVR	−0.5 (−1.5 to −0.3)	−0.5 (−1.3 to 3.2)	0.23

Patients who experienced > 50% reduction in the number of headache days per month were categorized as responders. All reported variables are continuous but not normally distributed, so medians and interquartile ranges were reported. The change in each parameter was calculated by subtracting the final value from the baseline value. *p*-values were calculated by Wilcoxon-Mann–Whitney test. MCA indicates middle cerebral artery. PCA indicates posterior cerebral artery. PI indicates pulsatility index. CVR indicates cerebrovascular reactivity.

## 4 Discussion

Our study found no major effect of 3–4 months of CGRP inhibitor therapy on interictal cerebral hemodynamics in individuals with migraine. In particular, dynamic measures of cerebrovascular function, CA and CVR, were unchanged after starting CGRP inhibitor therapy compared to baseline. The observed increase in posterior circulation PI may point to changes in downstream vascular tone, but this did not impede vascular responsiveness. Systemic hemodynamics were also unaffected, as quantified by blood pressure and peripheral vascular resistance. These findings provide some reassurance regarding the cerebrovascular safety of CGRP inhibitors, but it is important to recognize that the small sample size limited power to detect small but potentially clinically meaningful differences. Larger longitudinal studies with radiographic and clinically relevant endpoints would provide further certainty.

The relationship between CGRP and cerebral hemodynamics has been the focus of prior preclinical and clinical studies. Under normal physiologic conditions, CGRP influences cerebral autoregulation in the rodent brain (5), and intravenously administered CGRP induces an increase in CBFv in healthy individuals (6) and in individuals with migraine (7). Studies investigating CGRP antagonism provide further evidence of such a relationship. Preclinical models demonstrated that suffusion of murine cerebral surface with CGRP antibody serum severely blunted CA (8), while CGRP receptor desensitization results in attenuated compensatory vasodilation (5). However, the relationship between CGRP antagonism and cerebral hemodynamics remains controversial. Cerebral arteries are far more sensitive than systemic arteries to CGRP (22), but CGRP receptors may be largely limited to the abluminal side of the vessel (3, 23). Thus, CGRP targeting therapies may need to cross the blood brain barrier (BBB) to have a cerebrovascular effect (24), but the molecular size of monoclonal antibodies implies a limited ability to do so (10). On the other hand, mRNA for CGRP receptors has been identified within the endothelium of large cerebral vessels and the distal microvasculature (22), which raises the possibility that if this line of treatment has any cerebrovascular effect it may be via receptors on the luminal side or perhaps more indirectly. Though gepants are smaller than monoclonal antibodies, there is likely very little BBB penetration (25), but a preclinical study revealed that gepants affect the hemodynamics in the rodent brain (5). This may suggest more BBB penetration than initially anticipated, or perhaps again supports the notion that access to the abluminal side of the vessel is not essential. Another rodent model demonstrated that CGRP antagonists, including CGRP antibodies, opposed CGRP-induced dilatation after luminal administration, without the ability to cross the endothelium (12). This study also reported that another CGRP receptor antagonist studied (a large, hydrophilic peptide) inhibited abluminal CGRP even when perfused luminally (12). Taken together, these data raise the possibility that despite limited BBB penetration, anti-CGRP antibodies may directly or indirectly influence cerebral hemodynamics.

At the on-treatment follow-up visit, we did not observe an impact on cerebral hemodynamics in terms of CA, CVR, or CBFv across the entire cohort. However, patients who reported a > 50% decreases in headache frequency on-treatment, i.e., clinical responders, demonstrated a significant decrease in CBFv in both the MCA and the PCA. Given the small sample size, subgroups should be interpreted with caution, but this raises the possibility that some cerebral hemodynamic metrics may provide a physiologic biomarker of one’s clinical response to CGRP inhibitor therapy. This concept is similarly reflected in a recent TCD-based study which observed a significant change in CBFv in patients who experienced a good clinical response to CGRP antibody therapy (26). Not only was there a significant change after treatment, but those who responded well to the treatment had significantly lower baseline CBFv as compared to non-responders, further emphasizing the possibility that TCD may play a role in patient selection or monitoring drug effect (26). The lack of a control group (i.e., migraine patients not prescribed anti-CGRP therapies) in the current study presents a limitation, but baseline TCD characteristics in the current study, including flow velocity and PI, were comparable to previously reported TCD parameters in individuals with migraine (26). Importantly, the reported analysis did not rely on a control group because the treatment effect was quantified by a paired analysis (i.e., each patient’s baseline and follow-up data were compared to one another). This approach provided greater power and mitigated potential inter-subject variability. Still, inclusion of a control group would have facilitated more thorough cohort characterization.

While overall hemodynamics were preserved, we did observe an increase in posterior circulation PI. PI is a marker of downstream vascular resistance, which may be a result of an increase in vascular tone or a decrease in vessel diameter. CGRP is a potent vasodilator, so inhibition may impact measures of microvascular tone. The posterior circulation-specific finding with respect to PI is notable, as the posterior circulation is specifically involved in migraine pathophysiology. Posterior circulation CBF is increased in patients with migraine during the interictal period (27), and individuals who experience migraines have occipital hyperemia in response to visual stimuli (28). In migraine patients, posterior circulation blood flow is more sensitive to CGRP, and individuals who experience a headache after an intravenous infusion of CGRP have a particularly sensitive hemodynamic response (29). The changes in posterior circulation PI or CBFv may indicate potential biomarkers for clinical response, or they may play a more direct role in migraine prevention. These findings require validation in a larger cohort and further investigation to clarify the nature of the relationship between hemodynamics and clinical response.

Because CGRP is implicated in compensatory vasodilation, CVR, which quantifies the capacity of vasodilation (i.e., vascular reserve), is of particular interest. Reassuringly, CVR, as assessed by CO_2_ inhalation, was unaffected after the first few months of CGRP inhibition. This finding is in agreement with a recent study that observed no change in hypercapnia-induced vasodilation after starting erenumab (30). However, here we used inhaled 5% CO_2_ to quantify CVR, whereas the prior study relied on breath holding to induce hypercapnia, which is simple and effective but unfortunately achieves a highly variable degree of hypercapnia and may reduce PaO2 in a way that inadvertent effects the cerebral vasculature (31, 32). Despite these methodologic differences, these two cohorts support the idea that anti-CGRP antibodies do not negatively impact CO_2_-induced cerebral vasodilation.

Finally, CGRP may also impact systemic hemodynamics. CGRP has previously been shown to play a role in modulation of PVR and maintenance of basal arterial pressure (14, 33). In a cohort of patients with migraine, CGRP monoclonal antibody therapy resulted in a sustained increase in blood pressure, to the point where some required initiation of antihypertensive medications (34). Based on postmarketing data, there has been greater concern for elevated blood pressure in patients treated with erenumab specifically, which binds to the CGRP receptor, as opposed to the CGRP ligand (35). In fact, the FDA has amended the Warnings and Precautions section of the prescribing information for erenumab to include hypertension (36). In the current study, we did not observe a change in blood pressure, and no patients were started on antihypertensive medications during the course of follow-up, but these findings may be limited by the small sample size and limited duration of follow-up. Notably, our cohort did not include any patients in the final analysis who were treated with erenumab due to provider or insurance preference.

There are several limitations of this study. The small sample size limits power to detect small changes. This is particularly relevant seeing as no significant difference was noted in the primary analysis. A large cohort may be necessary to detect small but clinically relevant differences in the hemodynamic endpoints reported here. The small sample size also significantly limits generalizability given the relative diverse nature of individuals with migraine that could not be adequately reflected in this cohort. Subgroup analyses are particularly limited by the small sample size, and results should be interpreted cautiously. Rather, the subgroup observations reported here might justify future work that specifically explores the relationship between TCD metrics and treatment response in a prespecified fashion. Interpretation of this study is limited by the lack of a control group as previously noted. However, previous studies have reported stability of cerebral hemodynamics, including CA and CVR, over time in healthy individuals (37, 38), so a paired analysis, as was performed here, retains value without reliance on a control group. The cohort largely lacked vascular risk factors which further limits generalizability. CBFv was used as a surrogate for CBF, so changes should be interpreted cautiously when considering a therapy that may alter vascular diameter. However, CA and CVR are less hindered by this limitation, as they represent relative measures. Monitoring sessions were limited to resting state, so we cannot address the possibility of changes in cortical activation or response to metabolic demand, particularly in the posterior circulation. Lastly, while CA and CVR stability provides some reassurance, we cannot draw any conclusions regarding individual tolerance for ischemia and capacity for compensatory vasodilation in the context of CGRP inhibitor therapy.

In conclusion, CGRP inhibition for migraine prevention appears to have had little effect on cerebral hemodynamics. In particular, dynamic metrics of cerebrovascular health, CA and CVR, are preserved after 3 months of CGRP inhibitor treatment. However, the small sample provides limited power to recognize small but potentially meaningful differences which could be explored in a larger cohort. Those who have a favorable clinical response of CGRP inhibitor therapy (i.e., >50% reduction in migraine frequency) may have a more pronounced hemodynamic response, characterized as a small reduction in CBFv and an increase in PCA PI. These subgroup findings should be interpreted cautiously, but further investigation is warranted to confirm these observations in a larger cohort and clarify if cerebral hemodynamic changes are directly related to the therapeutic drug effect or if TCD metrics may serve as biomarkers of drug sensitivity.

## Data availability statement

The raw data supporting the conclusions of this article will be made available by the authors, without undue reservation.

## Ethics statement

The studies involving humans were approved by the University of Pennsylvania Institutional Review Board. The studies were conducted in accordance with the local legislation and institutional requirements. The participants provided their written informed consent to participate in this study.

## Author contributions

SC: Conceptualization, Methodology, Writing – original draft, Writing – review & editing, Data curation, Investigation. BC: Conceptualization, Formal analysis, Writing – original draft, Writing – review & editing. NR: Data curation, Methodology, Writing – original draft, Writing – review & editing. KH: Data curation, Investigation, Writing – original draft, Writing – review & editing. EK: Conceptualization, Formal analysis, Writing – original draft, Writing – review & editing. CF: Conceptualization, Formal analysis, Funding acquisition, Investigation, Methodology, Project administration, Resources, Writing – original draft, Writing – review & editing.
