# Supplementary material for: Effect of CGRP inhibitors on interictal cerebral hemodynamics in individuals with migraine
Source: Front Neurol. 2024 Apr 29;15:1399792. doi: 10.3389/fneur.2024.1399792 (PMC11091725; doi:10.3389/fneur.2024.1399792)
Supplement: Supplementary file 1 [file Data_Sheet_1.PDF]

Supplementary Material

Supplementary Table 1. Concomitant Medications

	**Baseline N=18**	**Follow-up N=18**
Migraine Prophylaxis		
Riboflavin	16%	16%
Magnesium	50%	50%
Metoprolol	11%	11%
Amitriptyline	5%	5%
Nortriptyline	5%	5%
Topiramate	11%	11%
OnabotulinumtoxinA	22%	22%
Other with potential cerebrovascular effect		
Triptan within 48 hours	11%	5%
Estrogen-containing OCP or estrogen replacement therapy	22%	22%
SSRI or SNRI	55%	55%

Categorical variables are reported as proportions.

Supplementary Table 2. Univariate analysis: association between baseline factors & hemodynamics

Dependent variable	Factors	Unadjusted coefficient	95% CI
Change in MCA Mx index	Age, per decade	-0.01	-0.07 – 0.06
	Male sex	0.01	-0.19 – 0.22
	White race	0.15	-0.09 – 0.39
	Baseline headache days/month	0.00	-0.01 – 0.01
	Hypertension	0.15	-0.13 – 0.43
	Hyperlipidemia	0.19	-0.04 – 0.41
	Diabetes	0.15	-0.24 – 0.55
	Active smoking	0.06	-0.10 – 0.22
	Blood pressure, per 10 mmHg	-0.02	-0.08 – 0.05
Change in PCA Mx index	Age, per decade	-0.02	-0.06 – 0.02
	Male sex	-0.05	-0.19 – 0.09
	White race	0.00	-0.17 – 0.17
	Baseline headache days/month	0.00	-0.01 – 0.01
	Hypertension	0.00	-0.20 – 0.20
	Hyperlipidemia	-0.05	-0.22 – 0.12
	Diabetes	0.11	-0.26 – 0.32
	Active smoking	0.00	-0.12 – 0.11
	Blood pressure, per 10 mmHg	-0.02	-0.06 – 0.02
Change in MCA CVR	Age, per decade	-0.26	-1.09 – 0.57
	Male sex	-0.15	-2.76 – 2.46
	White race	0.35	-2.76 – 3.47
	Baseline headache days/month	0.02	-0.11 – 0.14
	Hypertension	1.32	-2.30 – 4.94
	Hyperlipidemia	0.78	-2.31 – 3.87
	Diabetes	0.42	-4.63 – 5.46
	Active smoking	1.27	-0.69 – 3.23
	Blood pressure, per 10 mmHg	0.18	-0.66 – 1.03
Change in PCA CVR	Age, per decade	-0.15	0.45 – 0.52
	Male sex	-0.96	-3.65 – 1.73
	White race	1.19	-1.78 – 4.15
	Baseline headache days/month	-0.10	-0.21 – 0.02
	Hypertension	-2.53	-5.82 – 0.76
	Hyperlipidemia	-1.36	-4.31 – 1.58
	Diabetes	-0.50	-5.41 – 4.40
	Active smoking	-1.33	-3.22 – 0.56
	Blood pressure, per 10 mmHg	0.16	-0.55 – 0.86

Linear regression quantified the association between baseline factors and co-primary outcome measures. MCA indicates middle cerebral artery. PCA indicates posterior cerebral artery. CVR indicates cerebrovascular reactivity.

Supplementary Table 3. Comparing baseline characteristics between responders and non-responders

	**Responders n = 6**	**Non-responders n = 12**	**p-value**
Age, years	42 (27 – 52)	47 (25 – 67)	0.93
Sex, % female	83%	67%	0.44
Race, %			1.00
White	83%	83%	
Black or African American	17%	8%	
Asian	0%	8%	
Baseline migraine days per month	26 (15 – 30)	13 (9 – 23)	0.20
Medical history			
Hypertension	0%	17%	0.53
Type-2 diabetes	17%	0%	0.33
Hyperlipidemia	0%	25%	0.27
Coronary artery disease	0%	0%	1.0
Heart failure	0%	0%	1.0
Cigarette smoking, current	0%	8%	0.68

Age and migraine frequency are reported as median (interquartile range) given the small group sizes. Categorical variables are reported as proportions. P-values were calculated by Wilcoxon-Mann-Whitney test or Fisher's exact test for continuous and categorical variables, respectively.
